# Bioaccessibility of Phytochemicals, Amino Acids, Antioxidant Capacity, and Enzyme Inhibitory Activities of Hawthorn (*Crataegus* spp.) Fruits Following INFOGEST Simulated Gastrointestinal Digestion

**DOI:** 10.1002/fsn3.72188

**Published:** 2026-08-05

**Authors:** Kubra Feyza Erol, Gozde Kutlu, Hatice Bekiroglu, Osman Sagdıc, Fatih Tornuk

**Affiliations:** ^1^ Department of Nutrition and Dietetics, Hamidiye Faculty of Health Sciences University of Health Sciences İstanbul Türkiye; ^2^ Department of Gastronomy and Culinary Arts, Faculty of Fine Arts, Design and Architecture Ankara Medipol University Ankara Türkiye; ^3^ Department of Food Engineering Faculty of Agriculture, Sirnak University Sirnak Türkiye; ^4^ Department of Food Engineering, Faculty of Chemical and Metallurgical Engineering Yildiz Technical University, Davutpasa Campus İstanbul Türkiye; ^5^ Department of Nutrition and Dietetics, Faculty of Health Sciences Sivas Cumhuriyet University Sivas Türkiye

**Keywords:** bioactive potential, hawthorn fruit, in vitro antidiabetic activity, mineral content, static in vitro INFOGEST protocol

## Abstract

Hawthorn (*Crataegus* spp.) fruits are recognized as rich sources of phenolic compounds with well‐documented antioxidant and carbohydrate‐hydrolyzing enzyme inhibitory properties. However, the potential biological relevance of these phytochemicals depends largely on their stability and in vitro bioaccessibility during gastrointestinal digestion rather than on their native composition alone. This study comprehensively evaluated the impact of the standardized International Network of Food digestion (INFOGEST) model on the bioaccessibility of phenolic compounds and the associated changes in antioxidant capacity, free amino acid composition, and α‐amylase and α‐glucosidase inhibitory activities, while also characterizing the mineral composition of three *Crataegus* species. Simulated gastrointestinal digestion significantly decreased the bioaccessible fractions of total phenolics and flavonoids, accompanied by pronounced reductions in 2,2‐azino‐bis‐3‐ethylbenzothiazoline‐6‐sulfonic acid (ABTS), ferric reducing antioxidant power (FRAP), and cupric ion reducing antioxidant capacities (CUPRAC). Catechin was the predominant phenolic compound in all *Crataegus* species before digestion and remained the major phenolic in the bioaccessible fraction despite a substantial decrease during simulated gastrointestinal digestion. Although digestion attenuated the inhibitory activities against α‐amylase and α‐glucosidase, all hawthorn species retained appreciable enzyme inhibitory capacity following the intestinal phase. In parallel, l‐arginine, l‐glutamic acid, and l‐asparagine remained the predominant free amino acids in the bioaccessible fraction following simulated gastrointestinal digestion. In addition, distinct species‐dependent differences were observed in the mineral composition of the investigated hawthorn species. Collectively, these results demonstrate that simulated gastrointestinal digestion substantially influences the bioaccessibility of hawthorn phytochemicals and modulates their measured biological activities, whereas the investigated *Crataegus* species also differed in their nutritional composition. Overall, this study advances current understanding of the digestive stability and in vitro bioaccessibility of hawthorn phytochemicals and provides a scientific basis for selecting *Crataegus* species with favorable nutritional and functional attributes for future functional food development.

## Graphical abstract

Comprehensive compositional and functional characterization of three *Crataegus* species demonstrated that simulated gastrointestinal digestion substantially altered the bioaccessibility of phenolic compounds and associated biological activities. The pronounced interspecific variation observed highlights the importance of species selection for the development of hawthorn‐based functional food ingredients.

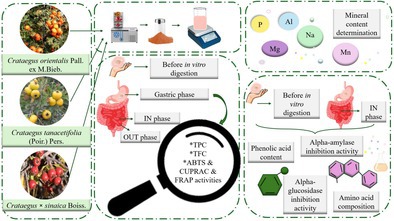

## 1 Introduction

The *Crataegus* genus (Rosaceae) comprises more than 280 species distributed primarily across the temperate regions of Europe, East Asia, and eastern North America (Abdel‐Rahman et al. 2022; El‐Hela et al. 2017; Refaat et al. 2010). Among these, *Crataegus sinaica* (Mount Sinai hawthorn) is a rare wild species that flowers between May and June and bears orange to red fruits. It has drawn attention for its economic importance and potential medicinal applications (Aly et al. 2025). In Türkiye, *Crataegus tanacetifolia* (Lam.) Pers. is an endemic species distinguished by its globose, yellow to reddish fruits approximately 2 cm in diameter (Güven et al. 2006). Similarly, *Crataegus orientalis* is commonly found in the Mediterranean region, Türkiye, and Iran, and is characterized by its dark yellow‐orange fruits, which are rich in amines, tannins, ascorbic acid, triterpene and flavone derivatives, and amygdins (Algül et al. 2024).

Hawthorn is widely recognized as a functional food ingredient and medicinal plant. Traditionally consumed fresh or processed into various food products, it has also been extensively used to support cardiovascular health. Owing to its rich phytochemical composition and associated health‐promoting properties, hawthorn has attracted increasing interest as a source of functional ingredients for food and nutraceutical applications (Zhang et al. 2022). These phytochemicals include flavonoids (0.1%–1%), oligomeric proanthocyanidins (1%–3%), triterpene acids (0.5%–1.4%), and organic acids (2%–6%), with flavonoids and proanthocyanidins being the primary contributors to their pharmacological activity (Benli et al. 2008). Phytochemical investigations have identified epicatechin, quercetin, hyperoside, vitexin‐2′′‐O‐rhamnoside, procyanidins B2 and C1, as well as ursolic acid in the leaves and fruits of various *Crataegus* species (El‐Hela et al. 2017). Moreover, additional compounds such as cholesterol, β‐sitosterol, uvaol, oleanolic acid, and multiple flavonoids have been isolated from the roots (El‐Hela et al. 2017). Traditionally, *Crataegus* species have been employed for their astringent and diuretic effects in the treatment of ailments such as diarrhea, menorrhagia, kidney disorders, and dropsy (Benli et al. 2008; Foster and Duke 1990). Beyond traditional uses, hawthorn has also gained clinical relevance as a well‐tolerated adjuvant in cardiovascular therapy agent, demonstrating improvements in heart failure symptoms, exercise capacity, and related physiological parameters (Edwards et al. 2012).

Food structure plays a pivotal role in regulating nutrient release, digestion kinetics, and metabolism, thereby influencing nutrient bioaccessibility and bioavailability (Mat et al. 2016). A thorough understanding of the interactions between food matrix and gastrointestinal digestion is essential for the development of functional foods and targeted nutritional strategies aimed at health promotion (Mat et al. 2016). Among various bioactive compounds, phenolics have received considerable attention due to their well‐documented health benefits. However, despite extensive literature supporting their biological activity, evaluating the bioaccessibility of phenolic compounds remains a critical challenge in food science (Eseberri et al. 2022). Because the concentration and composition of phenolic compounds vary considerably among foods, their dietary intake and physiological effects are also highly variable. Importantly, the biological effects of dietary phenolics depend not only on their concentration in the food matrix but also on their bioaccessibility—the fraction released from the food matrix during gastrointestinal digestion and available for intestinal absorption—and their subsequent bioavailability, defined as the fraction that reaches the systemic circulation following intestinal absorption and metabolic transformation (Hedren et al. 2002; Jiménez‐Pulido et al. 2024). Although in vivo studies are considered the gold standard for assessing nutrient bioaccessibility due to their physiological relevance, they are often constrained by ethical, logistical, and economic limitations (Mat et al. 2016; Rasera et al. 2023). To overcome these limitations, the International Network on Food Digestion (INFOGEST) developed a harmonized in vitro digestion model through international interdisciplinary collaboration. This protocol represents a major advancement in the field by providing a standardized, reproducible method for simulating gastrointestinal digestion under physiologically relevant conditions (Brodkorb et al. 2019; Rasera et al. 2023). The INFOGEST protocol includes sequential simulation of oral, gastric, and intestinal phases, incorporating parameters such as pH, enzyme activity, bile content, electrolyte concentrations, and digestion time to closely mimic the human digestive environment (Reboredo‐Rodríguez et al. 2021). Unlike previous studies, which primarily focused on total phenolic content and antioxidant activity, the present study provides a comprehensive assessment of hawthorn functionality by integrating (i) a comparative evaluation of three botanically distinct *Crataegus* species, (ii) mineral composition, (iii) amino acid profiling, (iv) changes in individual phenolic compounds, and (v) the bioaccessibility of antioxidant and antidiabetic activities following standardized INFOGEST digestion. To the best of our knowledge, such a comprehensive evaluation integrating these parameters under the standardized in vitro static INFOGEST protocol has not been previously reported for these hawthorn species. The central hypothesis of this study is that simulated gastrointestinal digestion significantly influences the bioaccessibility and biological activity of hawthorn phytochemicals in a species‐dependent manner. By adopting a harmonized methodological approach, this work seeks to generate reproducible and comparable data to better understand the role of digestion in modulating the functional properties of phenolic‐rich foods such as hawthorn. To address this knowledge gap, three botanically distinct *Crataegus* species were employed as a model system to elucidate species‐dependent differences in the digestive fate and bioaccessibility of bioactive compounds using the standardized INFOGEST static in vitro digestion model. By integrating comprehensive compositional characterization with harmonized in vitro digestion, this study provides a physiologically more relevant evaluation of hawthorn functionality than compositional analyses alone. Changes in phenolic composition, antioxidant capacity, free amino acid profiles, and α‐amylase and α‐glucosidase inhibitory activities were comprehensively evaluated before and after simulated gastrointestinal digestion, while the mineral composition of the investigated *Crataegus* species was characterized separately. The findings are expected to improve our understanding of how gastrointestinal digestion modulates the nutritional and functional attributes of hawthorn fruits and to provide evidence supporting future investigations of their application as functional food ingredients.

## 2 Materials and Methods

### 2.1 Material

The plant materials used in this study comprised the fruits of *Crataegus × sinaica* Boiss. (CS), *Crataegus tanacetifolia* (*Poir*.) *Pers*. (CT), and *Crataegus orientalis* *Pall. ex M. Bieb*. (CO), which were collected in October 2024 from Inkışla Village, located in the Gemerek District of Sivas Province, Türkiye (latitude: 39.348700, longitude: 36.033730, altitude: 1532 m). Botanical identification of the specimens was performed by Prof. Hüseyin Fakir (Faculty of Forestry, Isparta University of Applied Sciences, Türkiye) based on morphological characteristics. The harvested fruits were placed in polyethylene bags and transported to the laboratory under cooled conditions. Upon arrival, the samples were stored at +4°C until further processing. Prior to extraction, all plant materials were thoroughly washed with distilled water to remove surface contaminants, followed by freeze‐drying using a lyophilizer to preserve their bioactive components. The lyophilized samples were then ground into a fine powder using a knife mill and sieved through a 20‐mesh screen to obtain a uniform particle size.

### 2.2 Extract Preparation

To prepare the extracts, 5 g of dehydrated fruit was extracted with 50 mL of 80% (v/v) aqueous ethanol. This solvent system was selected because it has been widely reported to provide high extraction efficiency for a broad range of polar and moderately polar phenolic compounds while minimizing the extraction of non‐phenolic constituents. This mixture underwent continuous agitation at 150 rpm for 24 h at 25°C using a magnetic stirrer (Heidolph MR 3001K, Germany). Post‐extraction, the solution was subjected to centrifugation at 8000 rpm for 5 min (Multifuge X3 FR, Thermo Scientific, Gothenburg, Sweden). The collected supernatants were concentrated under reduced pressure using a rotary evaporator, followed by freeze‐drying (lyophilization) to obtain dry extracts. The lyophilized extracts were accurately weighed and reconstituted in 80% (v/v) aqueous ethanol to prepare stock solutions of known concentrations for subsequent biochemical analyses. The resulting extracts were then used for the quantification of total phenolic (TPC) and flavonoid (TFC) contents, as well as the assessment of 2,2‐azino‐bis‐3‐ethylbenzothiazoline‐6‐sulfonic acid (ABTS), ferric reducing antioxidant power (FRAP), and cupric ion reducing antioxidant capacities (CUPRAC), phenolic profile, mineral composition, and in vitro alpha glucosidase and alpha amylase inhibitory activities.

### 2.3 Mineral Composition

The determination of major (K, Mg, Na) and trace elements (Al, Mn) was conducted using Atomic Absorption Spectrometry (AAS) (Perkin Elmer Analyst 800, Norwalk, CT, USA), following the analytical procedures described by Erol, Kutlu, Olgun, and Tornuk (2025). For the solubilization of the mineral content, sample aliquots (approx. 0.3–1.0 g) underwent microwave‐assisted wet digestion. The digestion matrix consisted of 6.5 mL of 69% nitric acid (HNO_3_) and 1.5 mL of 30% hydrogen peroxide (H_2_O_2_). The thermal program was executed at 180°C and 220 psi, incorporating a ramp phase of 15–25 min followed by a 10–15 min isothermal hold. To ensure analytical accuracy and monitor potential contamination, reagent blanks were processed simultaneously under identical conditions. Upon completion of the digestion cycle, the vessels were cooled to ambient temperature, and the resulting solutions were diluted to a final volume of 50 mL using Milli‐Q ultrapure water. The concentration of each element was determined via external calibration using high‐purity standard stock solutions. The macro‐ and micro‐mineral constituents were calculated based on the linear regression data of the calibration curves and reported as mg per 100 g of sample.

### 2.4 In Vitro Bioaccessibility Tests

The in vitro bioaccessibility of hawthorn samples was evaluated using the standardized three‐stage simulated gastrointestinal digestion protocol proposed by Minekus et al. (2014). The influence of gastrointestinal digestion on TPC, TFC, ABTS radical scavenging, CUPRAC, and FRAP activities was examined by analyzing samples collected at each digestion step, including the undigested control. Additionally, the levels of phenolic compounds, as well as the inhibitory activities against α‐glucosidase and α‐amylase, were determined. In the corresponding tables, measurements obtained prior to digestion (for undigested sample) are reported as “before INFOGEST,” while values recorded after the intestinal (IN) phase are presented as “after INFOGEST,” representing the final stage of simulated digestion. During the gastric phase, samples were mixed with 8 mL of simulated gastric fluid, 5 μL of 0.3 M CaCl_2_·2H_2_O, and pepsin (2000 U/mL). The pH was adjusted to 3.0 using 3 M HCl, and the volume of acid added was noted. Subsequently, 1 mL of pepsin solution and additional CaCl_2_ were incorporated, and the final volume was adjusted to 20 mL with ultrapure water. The mixtures were incubated at 37°C for 2 h under continuous agitation. At the end of this stage, 4 mL aliquots were withdrawn from each sample for analysis. For the intestinal digestion step, the pH of the gastric digesta was raised to 7.0 using 1 M NaOH. Simulated intestinal fluid (8 mL), pancreatin (100 U mL^−1^), bile salts (10 mM), and 40 μL of 0.3 M CaCl_2_·2H_2_O were then added, and the total volume was adjusted to 40 mL with ultrapure water while maintaining a 1:1 (v/v) gastric‐to‐intestinal fluid ratio. Prior to intestinal digestion, the dialysis tubing (molecular weight cut‐off: 12 kDa) was thoroughly rinsed with 0.09% NaCl solution, filled with 0.1 M NaHCO_3_, sealed at both ends, and placed into centrifuge tubes. The digesta were then transferred into the dialysis tubing and incubated in a shaking water bath at 37°C for 2 h. Following digestion, the dialyzable (IN) fraction collected within the dialysis tubing and the non‐dialyzable (OUT) fraction were recovered separately, and 4 mL aliquots were collected for subsequent analyses. The dialyzable fraction was considered an estimate of the in vitro bioaccessible fraction, whereas changes observed throughout the gastric, IN, and OUT phases were interpreted as indicators of digestive stability. Because this approach is based on diffusion across a dialysis membrane, the IN fraction represents in vitro bioaccessibility only and should not be interpreted as intestinal absorption or in vivo bioavailability.

All post‐digestion samples obtained from the gastric and intestinal phases were centrifuged at 4000 rpm for 10 min. The resulting supernatants were filtered, transferred into 15 mL Falcon tubes, and stored at −4°C until further analysis. In the present study, the bioaccessibility index (BI) was defined as the proportion of compounds recovered in the dialyzable (IN) fraction relative to the total amount recovered in both dialyzable (IN) and non‐dialyzable (OUT) fractions following in vitro simulated gastrointestinal digestion (Equation 1).

(1) $$\mathrm{BI}\;\left(\%\right)=\frac{\text{IN phase}}{\text{IN phase}+\text{OUT phase}}\times 100$$

### 2.5 TPC

TPC was determined via a spectrophotometric method adapted from Kutlu (2024). All analyses were performed using stock solutions of freeze‐dried extracts prepared at a concentration of 10 mg/mL in 80% aqueous ethanol. In this procedure, 1 mL of the extract was combined with 0.5 mL of Folin–Ciocalteu reagent, followed by the addition of 2 mL of 7.5% (w/v) sodium carbonate after a 3‐min interval. The final volume was reached by adding distilled water to 10 mL. Following a 30‐min incubation in a dark environment, the absorbance of the mixture was measured at 760 nm using a UV–Vis spectrophotometer (UV‐1800, Shimadzu Corporation, Kyoto, Japan). Quantitative analysis was performed using a gallic acid standard curve (*y* = 12.734*x* − 0.2961, *R* ^2^ = 0.9938) prepared with concentrations ranging from 0.01 to 0.1 mg/mL. Results were recorded in triplicate and reported as mg gallic acid equivalents (GAE) per gram of extract (mean ± SD).

### 2.6 TFC

Following the protocol of Yasar et al. (2022), TFC was determined. All analyses were performed using stock solutions of freeze‐dried extracts prepared at a concentration of 10 mg/mL in 80% aqueous ethanol. In this assay, the reaction mixture was prepared by adding 0.3 mL of 5% (w/v) NaNO_2_ and 4 mL of distilled water to 1 mL of sample. Sequential additions of AlCl_3_ (0.3 mL) and 1 M NaOH (2 mL) were performed at 5 and 6 min, respectively. After adjusting the total volume to 10 mL with distilled water, the absorbance was measured at 510 nm against a blank using a UV–Vis spectrophotometer (UV‐1800, Shimadzu Corporation, Kyoto, Japan). Calculation of flavonoid levels was based on a catechin standard curve (*y* = 3.3432*x* + 0.0135, *R* ^2^ = 0.9992), and the final concentrations were expressed as mg CE/g dw.

### 2.7 ABTS Antioxidant Activity

The ABTS radical scavenging capacity of hawthorn extracts was assessed following the procedure reported by Erol, Kutlu, Icyer, and Tornuk (2025). Briefly, ABTS^+^ radicals were generated by mixing a 7 mM ABTS solution with 2.45 mM potassium persulfate and allowing the reaction to proceed in the dark at room temperature for 12–16 h. Prior to use, the resulting ABTS^+^ solution was diluted with distilled water until an absorbance value of 0.700 ± 0.02 at 734 nm was obtained. For the analysis, 100 μL of hawthorn extract solutions at varying concentrations (250–1250 μg/mL; calibration curve: *y* = 3.4411*x* + 0.0515, *R* ^2^ = 0.9979) were combined with the diluted ABTS^+^ solution in 2 mL microcuvettes. After incubation in the dark for 6 min, the decrease in absorbance was recorded at 734 nm using a UV–Vis spectrophotometer (UV‐1800, Shimadzu Corporation, Kyoto, Japan), and the radical scavenging activity was calculated accordingly.

### 2.8 The Ferric Reducing Antioxidant Power (FRAP) Assay

The FRAP of the samples was determined according to the principle of reducing ferric ions to their ferrous form under acidic conditions. Briefly, 100 μL of appropriately diluted extract was combined with 3.0 mL of freshly prepared FRAP reagent consisting of acetate buffer (300 mM, pH 3.6), 2,4,6‐tripyridyl‐s‐triazine (TPTZ) solution (10 mM in 40 mM HCl), and FeCl_3_·6H_2_O solution (20 mM). Following incubation at 37°C for 30 min, the absorbance of the reaction mixture was measured at 593 nm against a corresponding blank using a UV–Vis spectrophotometer (UV‐1800, Shimadzu Corporation, Kyoto, Japan). Antioxidant capacity was quantified using a calibration curve generated with Trolox standard solutions within the range of 100–1000 μmol/L, and the results were expressed as μmol trolox equivalent per gram of dry weight (μmol TE/g dw) (Kutlu 2024).

### 2.9 The Cupric Ion Reducing Antioxidant Capacity (CUPRAC) Assay

The CUPRAC of the samples was evaluated by measuring their ability to reduce Cu^2+^ ions in the presence of neocuproine. For this purpose, 0.5 mL of sample extract was mixed with 0.6 mL of distilled water and 3.0 mL of CUPRAC reagent prepared from equal volumes of CuCl_2_ solution (10 mM), ammonium acetate buffer (1 M), and neocuproine solution (7.5 mM). The resulting mixture was maintained at room temperature for 30 min to allow color development, after which the absorbance was recorded at 450 nm using a UV–Vis spectrophotometer (UV‐1800, Shimadzu Corporation, Kyoto, Japan). The antioxidant capacity of the samples was subsequently calculated based on the absorbance values obtained from the reaction system (Yasar et al. 2022).

### 2.10 Determination of Phenolic Acid Profiles by HPLC‐DAD

The concentrations of phenolic acid profile in hawthorn extracts were quantified both in their initial state and within the bioaccessible fraction (IN phase) obtained through the INFOGEST static in vitro digestion protocol. The chromatographic analysis was performed according to the methodology described by Yasar et al. (2022) with slight modifications. Quantitative analysis was carried out using a high‐performance liquid chromatography (HPLC) system equipped with an LC‐20 ad pump, an SPD‐M20A diode array detector (DAD), and a SIL‐20AHT autosampler. The separation was facilitated by a C18 Intersil ODS column (250 × 4.6 mm, 5 μm) maintained at a steady temperature of 40°C, with a flow rate of 1 mL/min. The separation of phenolic constituents was achieved using a binary solvent system consisting of 0.1% acetic acid in distilled water (Solvent A) and 0.1% acetic acid in acetonitrile (Solvent B). A flow rate of 1 mL/min was maintained throughout the following gradient program: 10% B from 0 to 2 min, increased to 30% B from 2 to 27 min, to 90% B from 27 to 50 min, to 100% B from 50 to 60 min, and then returned to initial conditions at 63 min. Identification of phenolic acid compounds was based on the comparison of their retention times with those of authentic standards. Quantification was performed using external standard calibration curves generated for each compound. The final results were reported as milligrams per gram of extract (mg/g extract).

### 2.11 In Vitro Methods Employed in Antidiabetic Studies

#### 2.11.1 Inhibition of Alpha Amylase Enzyme

The capacity of hawthorn extracts to inhibit alpha amylase was evaluated according to the protocol described by Erol, Kutlu, Icyer, and Tornuk (2025), with specific adjustments. Briefly, 50 μL of various extract concentrations were pre‐incubated with 25 μL of alpha amylase (0.5 U/mL) for 15 min at 37°C to facilitate enzyme‐inhibitor binding. Subsequently, the enzymatic reaction was initiated by introducing 50 μL of a 0.5% starch (solubilized in 20 mM phosphate buffer, pH 6.9). After an additional 10‐min incubation period at 37°C, the reaction was quenched by adding 600 μL of 3,5‐dinitrosalicylic acid (DNS) reagent. The mixture was then subjected to a 100°C heat treatment for 10 min. Upon cooling, the absorbance was measured at 540 nm using a microplate reader (Bio‐Tek, ELX808IU, USA). Acarbose served as the reference standard (positive control) for this assay. IC_50_ values were obtained from dose–response curves generated using at least eight extract concentrations (0.125, 0.25, 0.50, 0.75, 1.0, 1.5, 2.0, and 4.0 mg/mL) and calculated by nonlinear regression analysis.

#### 2.11.2 Inhibition of Alpha Glucosidases Enzyme

The alpha glucosidase inhibitory potential was determined through a microplate‐based assay. Initially, a 25 μL aliquot of the sample was combined with an equal volume of alpha glucosidase (0.5 U/mL) and allowed to equilibrate at 25°C for 10 min. The catalytic process commenced with the addition of 25 μL of 0.5 mM p‐nitrophenyl‐*α*‐D‐glucopyranoside as the substrate, followed by a 30‐min incubation phase at 37°C. To terminate the reaction, 100 μL of 0.2 M sodium carbonate was added to each well. The resulting chromogenic change was quantified by recording the absorbance at 405 nm using a microplate reader (Bio‐Tek, ELX808IU, USA) (Kutlu and Erol 2025). Moreover, IC_50_ values were determined from dose–response curves using nonlinear regression analysis based on at eight extract concentrations (0.125, 0.25, 0.50, 0.75, 1.0, 1.5, 2.0, and 4.0 mg/mL).

### 2.12 Determination of Amino Acid Composition

The quantification of amino acid derivatives was executed using a Shimadzu HPLC assembly, which integrated two LC‐9A solvent delivery pumps governed by an SCL‐6B system controller. Automated sample introduction was managed via a SIL‐6B auto‐injector. For the mobile phase preparation, an online Shodex DEGAS system (Tokyo, Japan) was utilized to ensure continuous degassing at a constant flow rate of 1.0 mL/min. Analyte resolution was achieved using an Inertsil ODS‐80A reversed‐phase column (150 × 4.6 mm i.d., 5 μm; GL Sciences, Tokyo, Japan). To ensure reproducibility, the column temperature was strictly maintained at 40°C employing a CTO‐6A thermostatic oven. Detection was performed with a Shimadzu RF‐550 fluorescence detector (12 μL flow cell capacity). The optical parameters were optimized based on the derivatizing agent used. The excitation and emission wavelengths were set at 490 and 530 nm for amino acid derivatives obtained with NBD‐PyNCS, and at 460 and 540 nm for those derived with DBD‐PyNCS. Peak integration and subsequent data processing were handled by a C‐R4A Chromatopac integrator. Quantitative assessment was facilitated through the construction of external calibration curves. These curves were generated by injecting standardized mixtures of 15 amino acids, specifically: l‐asparagine, l‐threonine, l‐serine, l‐glutamic acid, l‐proline, l‐glutamine, l‐methionine, l‐leucine, l‐tyrosine, l‐phenylalanine, l‐histidine, l‐lysine, l‐arginine, l‐valine, and l‐alanin (Toyo'oka and Liu 1995).

### 2.13 Statistical Analysis

All measurements were conducted in triplicate, and the results were expressed as mean ± standard deviation. Statistical analyses were performed using JMP 6 software (SAS Institute Inc., Cary, NC, USA). A one‐way analysis of variance (ANOVA) was applied, followed by Tukey's multiple comparison test to determine significant differences among groups at a 95% confidence level (*p* < 0.05).

## 3 Results and Discussion

### 3.1 Mineral Contents of Hawthorn Fruits

The mineral composition of the three *Crataegus* species is presented in Table 1. Potassium was the predominant mineral in all samples, followed by magnesium, sodium, aluminum, and manganese. Significant differences were observed among the species for all quantified minerals (*p* < 0.05). Among the investigated hawthorn species, CO contained the highest concentrations of potassium and sodium, whereas CS exhibited the highest levels of aluminum, magnesium, and manganese.

**TABLE 1:** Mineral content of undigested samples of various hawthorn species.

Sample name	Mineral content (mg/100 g)				
	K	Al	Mg	Na	Mn
CT	426.33 ± 7.37^C^	42.13 ± 0.47^C^	281.83 ± 1.71^B^	124.86 ± 0.59^C^	7.34 ± 0.08^C^
CO	485.00 ± 7.00^B^	48.39 ± 0.12^B^	240.21 ± 0.40^C^	154.33 ± 0.56^A^	9.67 ± 0.26^B^
CS	521.33 ± 10.50^A^	53.19 ± 0.41^A^	309.91 ± 1.62^A^	145.12 ± 0.70^B^	11.44 ± 0.15^A^

*Note:* Different uppercase letters (superscript A–C) within the same column denote statistically significant differences among hawthorn samples for a given mineral type (*p* < 0.05).

Abbreviations: CO, *Crataegus orientalis* ; CS, *Crataegus sinaica* Boiss.; CT, *Crataegus tanacetifolia* ; K, potassium; Ali aluminum; Mg, magnesium; Mn, manganese; Na, sodium.

The mineral concentrations observed in the present study are generally consistent with previous reports identifying potassium and magnesium as the major mineral constituents of hawthorn fruits (Özcan et al. 2005; Mironeasa and Todosi 2017). However, differences in the absolute concentrations among studies are expected and may reflect variations in species, geographical origin, soil composition, climatic conditions, fruit maturity, and analytical methodology. Similar observations were reported by Kutlu and Erol (2025), who emphasized that both environmental conditions and genetic factors play important roles in determining the mineral composition of plant‐derived foods. Overall, the results demonstrate that the investigated *Crataegus* species represent a valuable source of essential minerals, although their mineral profiles vary according to species‐specific and environmental factors.

### 3.2 TPC

The physiological effects of phenolic compounds are determined not solely by their dietary abundance but primarily by their release from the food matrix during digestion, which governs their bioaccessibility (Zhao et al. 2018). The TPC of the three *Crataegus* species differed significantly before digestion (*p* < 0.05), with CS exhibiting the highest value (15.33 mg GAE/g dw), followed by CO (14.01 mg GAE/g dw) and CT (11.29 mg GAE/g dw). Following INFOGEST simulated digestion, TPC levels decreased significantly in both the gastric and intestinal phases compared with the undigested samples (*p* < 0.05). These differences are consistent with previous reports demonstrating considerable interspecific variation in phenolic accumulation among *Crataegus* species, which has been attributed to genetic background, environmental conditions, and fruit maturity (Tomás‐Barberán and Espín 2001). During the gastric phase, a pronounced reduction in TPC was observed in all samples, with values decreasing by approximately 78.12%, 59.81%, and 77.43% for CT, CO, and CS, respectively. This decline is likely associated with the instability and partial degradation of extracted phenolic compounds under digestive conditions (Liu et al. 2021). Interestingly, TPC partially recovered during the intestinal phase, particularly in CT and CS, suggesting the release of phenolic compounds previously bound to the food matrix under alkaline conditions. Similar observations have been reported in previous in vitro digestion studies, where matrix disruption and enzymatic hydrolysis enhanced the liberation of bound phenolics (Zheng et al. 2018; Pollini et al. 2022). Pollini et al. (2022) suggested that hydrolysis of phenolic glycosides into their corresponding aglycones may also contribute to the increased recoverable phenolics during digestion. Furthermore, interactions between polyphenols and dietary constituents may influence their release by modifying molecular weight, solubility, and structural accessibility (Karaś et al. 2017; Spínola et al. 2018).

In the OUT phase, CO exhibited the highest TPC (5.69 mg GAE/g dw), closely followed by CS (5.45 mg GAE/g dw), whereas CT showed the lowest value (3.10 mg GAE/g dw). These findings indicate that a proportion of phenolic compounds was transferred to the OUT fraction for CO and CS samples following simulated digestion, although the recovered amounts were considerably lower than those in the undigested samples. A substantial portion of phenolic compounds remained in the non‐absorbed fraction during simulated digestion, with limited transfer to the bioaccessible phase. Gonçalves et al. (2018) also reported that although some phenolics may pass through intestinal models, a considerable fraction remains unabsorbed due to limited permeability and structural constraints. The BI values ranged from 35.94% to 54.39%, with CT exhibiting the highest bioaccessibility despite having the lowest initial TPC, whereas CO showed the highest initial phenolic content but the lowest BI. This finding demonstrates that a higher initial phenolic concentration does not necessarily result in greater bioaccessibility, as phenolic stability and release depend on molecular structure, degree of polymerization, and interactions with dietary macromolecules (Zhao et al. 2018; Tomé‐Sánchez et al. 2021).

Previous studies likewise reported that only a fraction of phenolic compounds becomes bioaccessible after gastrointestinal digestion, whereas a considerable proportion remains in the non‐absorbed fraction because of limited permeability and structural constraints (Fazzari et al. 2008; Gonçalves et al. 2018; Erol et al. 2024; Erol and Kutlu 2025). Zhao et al. (2018) further demonstrated that free phenolics generally exhibit higher bioaccessibility than bound phenolics because they are readily released in the upper gastrointestinal tract, while bound forms require additional enzymatic and microbial degradation in the colon. The observed differences among the three *Crataegus* species may also reflect variations in genotype and growing conditions. García‐Mateos et al. (2013) demonstrated that environmental stresses, such as drought and low temperature, enhance polyphenol accumulation in *C. laevigata* and *C. monogyna* by stimulating phenylpropanoid metabolism, whereas Chen et al. (2019) highlighted the additional influence of altitude, light intensity, temperature, soil nutrients, and fruit maturity on phenolic composition. Our findings are generally consistent with those of Ozkan et al. (2023), who reported considerable species‐dependent differences in the bioaccessibility of hawthorn phenolics following simulated gastrointestinal digestion. In their study, TPC bioaccessibility ranged from 16% in *C. monogyna* to 61% in different *C. orientalis* samples, supporting the present observation that the digestive stability and bioaccessibility of phenolic compounds are highly dependent on species characteristics and food matrix composition. Taken together, these findings demonstrate that the in vitro bioaccessibility of hawthorn phenolics is influenced not only by their initial abundance but also by species‐specific differences in phenolic composition and digestive stability during simulated gastrointestinal digestion.

### 3.3 TFC

Flavonoids, produced via the shikimate pathway, play important physiological roles in plants and have been associated with antimicrobial, antioxidant, hypotensive, and anti‐cancer effects (Edwards et al. 2012). Because flavonoids undergo extensive structural modifications during digestion, their physiological relevance depends largely on their bioaccessibility rather than their initial concentration (Zhang et al. 2024). Before digestion, CS contained the highest flavonoid concentration (1.98 mg CE/g dw), while CT and CO exhibited significantly lower values (1.61 and 1.70 mg CE/g dw, respectively) (*p* < 0.05). Gastric digestion caused a marked reduction in TFC in all species, with reductions exceeding ~96%, indicating the susceptibility of flavonoids to acidic conditions and digestive enzymes. A slight increase was observed during the intestinal phase, particularly in CS, which may be attributed to the release of flavonoids previously associated with the food matrix or to structural transformations occurring during digestion. Nevertheless, flavonoid concentrations remained substantially lower than their initial values throughout the digestion process. The OUT fraction contained only 0.11–0.15 mg CE/g dw of flavonoids, corresponding to BI values ranging from 43.20% to 49.13%. The relatively moderate flavonoid bioaccessibility observed in the present study agrees with previous findings indicating that flavonoids are particularly sensitive to gastrointestinal conditions because of oxidation, deglycosylation, and structural rearrangements (Oteiza et al. 2018). Furthermore, structural conversion of flavonoids into smaller phenolic metabolites during digestion may reduce their detection by the aluminum chloride colorimetric assay, thereby contributing to the lower measured TFC values. Similar observations were reported by Zheng et al. (2018), who demonstrated that flavonoids undergo considerable structural changes and redistribution throughout gastrointestinal digestion.

The observed differences among the three *Crataegus* species may also reflect genetic and environmental variability. Chen et al. (2019) reported that flavonoid accumulation is strongly influenced by species, geographical origin, environmental conditions, and fruit maturity. In addition, Edwards et al. (2012) described quercetin‐3‐*O*‐galactoside, vitexin‐2″‐*O*‐rhamnoside, and acetylvitexin‐2″‐*O*‐rhamnoside as the major flavonoids in *Crataegus*, highlighting the structural diversity that may contribute to differences in digestive stability. Lima et al. (2019) similarly attributed the reduction in flavonoid content after simulated digestion to the physicochemical conditions of the gastrointestinal tract, whereas Zhang et al. (2024) emphasized that interactions with proteins, lipids, carbohydrates, and dietary fiber can either limit or enhance flavonoid bioaccessibility. Our results are generally consistent with those of Ozkan et al. (2023), who reported pronounced species‐dependent differences in flavonoid bioaccessibility among hawthorn fruits following simulated gastrointestinal digestion. Their findings support the present observation that digestive stability and flavonoid bioaccessibility vary considerably according to species characteristics and food matrix composition. The diverse phytochemical composition of hawthorn fruits, including flavonoids, phenolic acids, and other secondary metabolites, may further contribute to the differences observed in flavonoid recovery following digestion (Nazhand et al. 2020).

### 3.4 Antioxidant Activities

The antioxidant capacities of the three *Crataegus* species, determined by the ABTS, FRAP, and CUPRAC assays before and after simulated gastrointestinal digestion, are presented in Table 2. All antioxidant assays demonstrated that digestion significantly affected the antioxidant properties of the hawthorn extracts (*p* < 0.05), although the magnitude of the changes varied according to species and digestion stage.

**TABLE 2:** TPC, TFC, ABTS, FRAP, and CUPRAC antioxidant activities as well as bioaccessibility index (BI) of various hawthorn species during the INFOGEST in vitro gastrointestinal digestion process (undigested, oral, gastric, intestinal‐IN, and intestinal‐OUT phases).

Type of analysis	Simulated digestion stages	CT	CO	CS
TPC	Undigested sample (mg GAE/g in dw)	11.29 ± 0.12^C^	14.01 ± 0.18^B^	15.33 ± 0.05^A^
	Gastric‐phase (mg GAE/g in dw)	2.47 ± 0.04^C^	5.63 ± 0.06^A^	3.46 ± 0.05^B^
	IN‐phase (mg GAE/g in dw)	3.70 ± 0.03^B^	3.19 ± 0.11^C^	4.58 ± 0.05^A^
	OUT‐phase (mg GAE/g in dw)	3.10 ± 0.02^C^	5.69 ± 0.05^A^	5.45 ± 0.16^B^
	BI (%)	54.39 ± 0.32^A^	35.94 ± 0.86^C^	45.65 ± 0.45^B^
TFC	Undigested sample (mg CE/g in dw)	1.61 ± 0.10^B^	1.70 ± 0.04^B^	1.98 ± 0.03^A^
	Gastric‐phase (mg CE/g in dw)	0.07 ± 0.00^B^	0.09 ± 0.00^A^	0.08 ± 0.00^B^
	IN‐phase (mg CE/g in dw)	0.09 ± 0.00^B^	0.11 ± 0.01^B^	0.13 ± 0.01^A^
	OUT‐phase (mg CE/g in dw)	0.12 ± 0.01^B^	0.11 ± 0.01^B^	0.15 ± 0.01^A^
	BI (%)	43.20 ± 2.23^B^	49.13 ± 1.41^A^	46.51 ± 0.76^AB^
ABTS activity	Undigested sample (mg GAE/g in dw)	23.07 ± 0.86^C^	27.43 ± 1.31^B^	34.55 ± 0.74^A^
	Gastric‐phase (mg GAE/g in dw)	4.62 ± 0.11^B^	6.77 ± 0.07^A^	4.73 ± 0.10^B^
	IN‐phase (mg GAE/g in dw)	6.82 ± 0.09^B^	7.24 ± 0.06^A^	7.19 ± 0.06^A^
	OUT‐phase (mg GAE/g in dw)	11.72 ± 0.16^A^	10.39 ± 0.07^B^	10.35 ± 0.11^B^
	BI (%)	36.80 ± 0.21^B^	41.05 ± 0.33^A^	40.97 ± 0.07^A^
FRAP activity	Undigested sample (μmol TE/g in dw)	32.88 ± 0.32^A^	21.58 ± 0.23^C^	28.40 ± 0.20^B^
	Gastric‐phase (μmol TE/g in dw)	17.22 ± 0.33^A^	11.34 ± 0.05^C^	15.09 ± 0.50^B^
	IN‐phase (μmol TE/g in dw)	9.82 ± 0.09^A^	9.33 ± 0.13^B^	5.11 ± 0.13^C^
	OUT‐phase (μmol TE/g in dw)	12.36 ± 0.33^B^	16.59 ± 0.13^A^	7.45 ± 0.11^C^
	BI (%)	44.28 ± 0.81^A^	35.99 ± 0.18^C^	40.68 ± 0.93^B^
CUPRAC activity	Undigested sample (μmol TE/g in dw)	78.43 ± 0.04 ^A^	43.75 ± 0.07^C^	68.19 ± 0.33^B^
	Gastric‐phase (μmol TE/g in dw)	38.04 ± 1.16 ^A^	22.84 ± 0.16^C^	31.74 ± 0.58^B^
	IN‐phase (μmol TE/g in dw)	12.21 ± 0.73^B^	8.58 ± 0.28^C^	13.70 ± 0.20^A^
	OUT‐phase (μmol TE/g in dw)	24.66 ± 0.17^A^	15.49 ± 0.21^C^	18.71 ± 0.23^B^
	BI (%)	55.69 ± 0.95^A^	35.63 ± 0.97^C^	42.28 ± 0.63^B^

*Note:* Different uppercase letters within the same line indicate significant differences between the hawthorn samples (*p* < 0.05), whereas identical letters indicate no statistically significant difference (*p* > 0.05).

Abbreviations: BI, bioaccessibility index; CE, catechin equivalent; CO, *Crataegus orientalis* ; CS, *Crataegus sinaica* Boiss.; CT, *Crataegus tanacetifolia* ; dw, dry weight; GAE, gallic acid equivalent; TE, Trolox equivalent; TFC, total flavonoid content; TPC, total phenolic content.

Before digestion, ABTS radical scavenging activity differed significantly among the species, with CS exhibiting the highest activity, followed by CO and CT. A marked reduction was observed during the gastric phase, whereas partial recovery occurred during the intestinal and OUT phases. Nevertheless, ABTS values remained substantially lower than those of the undigested samples throughout digestion. The partial recovery observed after the gastric phase may reflect the release of antioxidant compounds previously associated with the food matrix or structural modifications occurring during digestion. Similar trends were observed for the reducing antioxidant capacity determined by the FRAP and CUPRAC assays. Both assays showed pronounced decreases following gastric digestion, indicating that gastrointestinal conditions altered the redox‐active compounds responsible for electron transfer (Apak et al. 2016). Although a slight recovery was observed in the fractions, particularly for CT in the CUPRAC and FRAP assays, the antioxidant capacities remained below their initial values. Among the investigated species, CT exhibited the highest BI for both FRAP and CUPRAC, suggesting greater stability of its reducing antioxidant constituents during simulated digestion.

The reductions observed in ABTS, FRAP, and CUPRAC activities closely paralleled the decline in total phenolic content, supporting the major contribution of phenolic compounds to the antioxidant capacity of hawthorn fruits. However, the magnitude of the decrease differed among the antioxidant assays, indicating that antioxidant activity depends not only on the concentration of phenolic compounds but also on their chemical structure, redox properties, and interactions with the food matrix (Čeryová et al. 2026). Similar observations have been reported by Yu et al. (2021), who demonstrated that the antioxidant response of phenolic compounds depends on both their structural characteristics and the analytical method employed.

Previous studies have suggested that interactions between phenolic compounds and proteins, carbohydrates, and other macromolecules may limit the release and dialyzability of antioxidants during gastrointestinal digestion (Pasli et al. 2019; Tomé‐Sánchez et al. 2021). In addition, structural transformations, including hydrolysis of phenolic glycosides and the release of lower‐molecular‐weight compounds, may modify antioxidant responses measured by different assays (de Morais et al. 2020; Tang et al. 2023). These mechanisms may explain the partial recovery of antioxidant activity observed during the later stages of digestion. Our findings differ from those reported by Ozkan et al. (2023), who observed increases in CUPRAC values following simulated digestion in several *Crataegus* species. Such discrepancies may be attributed to differences in species, phenolic composition, fruit maturity, extraction procedures, and matrix characteristics, all of which can influence the stability and release of antioxidant compounds during gastrointestinal digestion. Overall, the present results indicate that simulated digestion substantially modifies the antioxidant capacity of hawthorn fruits, while the extent of these changes is highly species dependent.

### 3.5 Phenolic Profile of Hawthorn Fruits Before and After Gastrointestinal Digestion

The individual phenolic profiles of the three *Crataegus* species before and after simulated gastrointestinal digestion are presented in Table 3. Eight phenolic compounds—protocatechuic acid, catechin, chlorogenic acid, caffeic acid, *p*‐coumaric acid, rutin, ferulic acid, and quercetin—were identified, whereas gallic acid, 4‐hydroxybenzoic acid, syringic acid, ellagic acid, *o*‐coumaric acid, chrysin, myricetin, kaempferol, and sinapic acid were not detected. Catechin was the predominant phenolic compound in all three *Crataegus* species before digestion, followed by chlorogenic acid and protocatechuic acid. CS exhibited the highest catechin concentration, whereas CT contained the greatest amount of chlorogenic acid. Ferulic acid and caffeic acid were also present in appreciable quantities, while rutin, quercetin, and *p*‐coumaric acid were detected at comparatively lower concentrations. These results agree with previous reports identifying catechin, caffeic acid, and rutin as the major phenolic constituents of hawthorn fruits (Muradoğlu et al. 2019). Simulated gastrointestinal digestion significantly affected the phenolic profile of all three species (*p* < 0.05). The concentrations of all identified phenolic compounds decreased after digestion, with catechin exhibiting the greatest absolute reduction. Despite this decline, catechin remained the predominant phenolic constituent in the bioaccessible fraction, indicating greater digestive stability than the other identified phenolics. Similar decreases were observed for chlorogenic acid, caffeic acid, protocatechuic acid, ferulic acid, rutin, quercetin, and *p*‐coumaric acid, demonstrating that both phenolic acids and flavonoids are susceptible to gastrointestinal conditions.

**TABLE 3:** The individual phenolic profile of various hawthorn species in undigested samples and IN phase of simulated gastrointestinal digestion.

Types of phenolic acids, mg/g	CT		CO		CS	
	Undigested sample	IN phase	Undigested sample	IN phase	Undigested sample	IN phase
Protocatechuic acid	6.84 ± 0.01^A,b^	2.45 ± 0.01^B,b^	7.25 ± 0.01^A,a^	3.62 ± 0.01^B,a^	4.28 ± 0.01^A,c^	2.18 ± 0.01^B,c^
Catechin	22.56 ± 0.01^A,b^	6.82 ± 0.01^B,c^	18.47 ± 0.01^A,c^	7.85 ± 0.01^B,b^	27.94 ± 0.0 1^A,a^	11.33 ± 0.01^B,a^
Chlorogenic acid	17.83 ± 0.01^A,a^	4.93 ± 0.01^B,a^	8.94 ± 0.01^A,c^	2.25 ± 0.01^B,c^	11.46 ± 0.01^A,b^	3.86 ± 0.01^B,b^
Caffeic acid	5.73 ± 0.01^A,b^	1.46 ± 0.01^B,b^	6.83 ± 0.01^A,a^	1.87 ± 0.01^B,a^	3.46 ± 0.01^A,c^	1.07 ± 0.01^B,c^
*p*‐Coumaric acid	1.45 ± 0.01^A,c^	0.36 ± 0.01^B,c^	2.46 ± 0.01^A,b^	1.14 ± 0.01^B,a^	2.78 ± 0.01^A,a^	0.92 ± 0.01^B,b^
Rutin	1.54 ± 0.01^A,a^	0.72 ± 0.01^B,a^	0.80 ± 0.01^A,c^	0.31 ± 0.01^B,c^	1.34 ± 0.01^A,b^	0.57 ± 0.01^B,b^
Ferulic acid	5.73 ± 0.01^A,a^	2.65 ± 0.01^B,a^	3.52 ± 0.01^A,b^	1.67 ± 0.01^B,b^	2.56 ± 0.01^A,c^	1.09 ± 0.01^B,c^
Quercetin	0.46 ± 0.01^A,c^	0.31 ± 0.01^B,b^	0.92 ± 0.01^A,a^	0.43 ± 0.01^B,a^	0.68 ± 0.01^A,b^	0.25 ± 0.01^B,c^
Gallic acid	N.D.	N.D.	N.D.	N.D.	N.D.	N.D.
4‐Hydroxybenzoic acid	N.D.	N.D.	N.D.	N.D.	N.D.	N.D.
Syringic acid	N.D.	N.D.	N.D.	N.D.	N.D.	N.D.
Ellagic acid	N.D.	N.D.	N.D.	N.D.	N.D.	N.D.
*o*‐Coumaric acid	N.D.	N.D.	N.D.	N.D.	N.D.	N.D.
Chrysin	N.D.	N.D.	N.D.	N.D.	N.D.	N.D.
Myricetin	N.D.	N.D.	N.D.	N.D.	N.D.	N.D.
Kaempferol	N.D.	N.D.	N.D.	N.D.	N.D.	N.D.
Sinapic acid	N.D.	N.D.	N.D.	N.D.	N.D.	N.D.

*Note:* Different uppercase letters (superscript A, B) within the same line denote statistically significant differences among undigested sample and IN phase for each hawthorn sample (*p* < 0.05). Different lowercase letters within the same digestion phase (undigested or IN phase) indicate significant differences among Crataegus species (*p* < 0.05).

Abbreviations: CO, *Crataegus orientalis* ; CS, *Crataegus sinaica* Boiss.; CT, *Crataegus tanacetifolia* ; N.D., not detected.

The overall reduction in individual phenolics was consistent with the decline in TPC (Table 2), suggesting that gastrointestinal digestion promotes structural modification, oxidation, hydrolysis, and interactions with digestive constituents, thereby reducing the recovery of native phenolic compounds (Alminger et al. 2014). Previous studies have likewise demonstrated that protein–polyphenol, fiber–polyphenol, and polysaccharide–polyphenol interactions may limit the release and dialyzability of phenolic compounds while simultaneously improving their structural stability during digestion (Palafox‐Carlos et al. 2011; Zhang, Yan, et al. 2020; Shahidi and Pan 2022). In addition, oxidative reactions and structural transformations occurring throughout digestion may further contribute to the lower concentrations detected after digestion (Wojtunik‐Kulesza et al. 2020). Species‐dependent differences in phenolic stability were also evident. Although CO exhibited the highest initial protocatechuic acid concentration, relatively greater recovery was observed after digestion. Likewise, CS retained the highest catechin concentration, whereas CT showed greater retention of chlorogenic acid, ferulic acid, rutin, and quercetin. These findings indicate that digestive stability depends not only on the initial abundance of individual phenolics but also on species‐specific differences in matrix composition and molecular interactions that influence phenolic release during digestion (Zengin et al. 2025).

The present results are generally consistent with previous studies reporting considerable variation in the phenolic composition of *Crataegus* species (Zhang et al. 2001; Muradoğlu et al. 2019; Şeker et al. 2025). In particular, differences in rutin and quercetin contents among hawthorn species have been attributed to both genetic and environmental factors. Moreover, the substantial reductions observed for these flavonoids after digestion agree with previous reports demonstrating that flavonoid glycosides readily undergo hydrolysis, oxidation, and complex formation with dietary macromolecules during gastrointestinal digestion, thereby reducing their measurable concentrations (Dima et al. 2020; Shahidi and Pan 2022). It should also be considered that the dialysis model estimates only the dialyzable fraction of phenolic compounds and therefore may underestimate the physiological fate of high‐molecular‐weight or matrix‐bound phenolics (Wojtunik‐Kulesza et al. 2020). Such compounds may remain in the non‐dialyzable fraction and subsequently undergo microbial metabolism in the colon, generating lower‐molecular‐weight metabolites with biological relevance (Koudoufio et al. 2020; Kwofie and Orsat 2026). Accordingly, the present findings reflect phenolic bioaccessibility under simulated gastrointestinal conditions rather than in vivo conditions.

### 3.6 Antidiabetic Activity

The inhibitory activities of the three *Crataegus* species against α‐amylase and α‐glucosidase before and after simulated gastrointestinal digestion are presented in Table 4. For both enzymes, all extracts exhibited concentration‐dependent inhibitory activity, with inhibition increasing as extract concentration increased. Before digestion, CT exhibited the strongest α‐amylase inhibitory activity, showing the lowest IC_50_ value among the hawthorn species and an inhibitory potency comparable to that of acarbose. In contrast, CS and CO showed significantly weaker inhibition. Following simulated gastrointestinal digestion, α‐amylase inhibitory activity decreased in all samples, as reflected by increased IC₅₀ values. Nevertheless, measurable inhibitory activity was retained after digestion, indicating that part of the inhibitory constituents remained stable under simulated gastrointestinal conditions. Among the digested samples, CO showed the greatest α‐amylase inhibitory activity, whereas CS exhibited the lowest.

**TABLE 4:** In vitro α‐amylase and α‐glucosidase inhibitory activities of various hawthorn species in the undigested samples and IN phase of simulated gastrointestinal digestion.

Enzyme type	Digestion status	Sample namee	Inhibition percentages								
			0.125 μg/mL	0.25 μg/mL	0.50 μg/mL	0.75 μg/mL	1 μg/mL	1.5 μg/mL	2 μg/mL	4 μg/mL	IC_50_ (μg/mL)
Alpha‐amylase	Undigested sample	CT	8.97 ± 0.25^H^	15.97 ± 0.42^G^	28.00 ± 0.62^F^	37.97 ± 0.85^E^	44.97 ± 0.85^D^	54.00 ± 0.95^C^	59.10 ± 0.75^B^	76.03 ± 0.80^A^	1.17
		CO	5.00 ± 0.26^H^	10.00 ± 0.26^G^	18.03 ± 0.60^F^	27.10 ± 0.75^E^	33.03 ± 0.80^D^	39.00 ± 0.80^C^	43.70 ± 0.66^B^	63.00 ± 0.80^A^	2.29
		CS	7.03 ± 0.25^G^	13.10 ± 0.30^F^	24.03 ± 0.47^E^	34.00 ± 0.75^D^	40.93 ± 0.80^C^	48.03 ± 0.80^B^	50.20 ± 0.66^B^	71.00 ± 0.80^A^	2.01
		Acarbose	10.07 ± 0.25^H^	18.00 ± 0.46^G^	30.93 ± 0.70^F^	42.07 ± 0.75^E^	48.93 ± 0.80^D^	55.13 ± 0.80^C^	58.37 ± 0.65^B^	82.03 ± 0.80^A^	1.19
	IN phase	CT	0.67 ± 0.07^H^	2.35 ± 0.24^G^	4.90 ± 0.97^F^	7.30 ± 0.10^E^	11.54 ± 0.15^D^	18.75 ± 0.24^C^	27.62 ± 0.40^B^	59.35 ± 0.83^A^	3.26
		CO	0.16 ± 0.02^H^	0.71 ± 0.07^G^	1.76 ± 0.08^F^	4.46 ± 0.13^E^	9.10 ± 0.26^D^	13.61 ± 0.38^C^	13.16 ± 0.36^B^	33.32 ± 1.29^A^	2.36
		CS	0.38 ± 0.03^G^	0.91 ± 0.04^G^	2.40 ± 0.17^F^	5.72 ± 0.08^E^	9.45 ± 0.19^D^	13.53 ± 0.17^C^	22.97 ± 0.33^B^	42.24 ± 0.34^A^	5.15
		Acarbose	5.70 ± 0.06^G^	7.48 ± 0.12^F^	15.49 ± 0.23^E^	22.36 ± 0.20^D^	29.65 ± 0.18^C^	30.38 ± 0.23^C^	35.62 ± 0.55^B^	65.11 ± 0.77^A^	2.52
Alpha‐glucosidase	Undigested sample	CT	3.35 ± 0.09^H^	7.59 ± 0.22^G^	18.32 ± 0.18^F^	29.58 ± 0.24^AE^	37.04 ± 0.26^D^	42.73 ± 0.82^C^	59.80 ± 0.61^B^	78.41 ± 3.42^A^	1.69
		CO	1.27 ± 0.02^H^	2.12 ± 0.08^G^	6.08 ± 0.18^F^	14.75 ± 0.10^E^	19.59 ± 0.14^D^	29.38 ± 0.19^C^	42.77 ± 2.23^B^	63.00 ± 0.80^A^	2.36
		CS	2.34 ± 0.08^H^	4.65 ± 0.07^G^	9.35 ± 0.09^F^	19.89 ± 0.19^E^	26.29 ± 0.09^D^	35.64 ± 0.23^C^	51.91 ± 2.37^B^	71.00 ± 0.80^A^	1.92
		Acarbose	8.55 ± 2.87^G^	8.43 ± 0.05^G^	18.00 ± 0.13^F^	25.78 ± 0.13^E^	33.48 ± 0.20^D^	55.55 ± 0.34^C^	63.59 ± 0.16^B^	82.03 ± 0.80^A^	1.39
	IN phase	CT	3.17 ± 0.28^H^	5.83 ± 0.35^G^	10.40 ± 0.45^F^	14.22 ± 0.39^E^	17.35 ± 0.45^D^	22.90 ± 0.46^C^	27.64 ± 0.12^B^	42.49 ± 0.14^A^	5.08
		CO	2.08 ± 0.23^H^	4.20 ± 0.30^G^	7.37 ± 0.28^F^	10.18 ± 0.33^E^	12.58 ± 0.43^D^	16.38 ± 0.43^C^	19.36 ± 0.07^B^	24.35 ± 0.09^A^	13.56
		CS	1.80 ± 0.30^H^	3.83 ± 0.40^G^	6.50 ± 0.60^F^	9.20 ± 0.60^E^	12.07 ± 0.65^D^	16.77 ± 0.65^C^	21.28 ± 0.12^B^	44.88 ± 0.34^A^	4.46
		Acarbose	8.55 ± 2.87^G^	8.43 ± 0.05^G^	18.00 ± 0.13^F^	25.78 ± 0.13^E^	33.48 ± 0.20^D^	55.55 ± 0.34^C^	63.59 ± 0.16^B^	82.03 ± 0.80^A^	1.39

*Note:* Differences among hawthorn concentrations in enzyme inhibition percentages are indicated by different capital letters within the same line, with a significance level of *p* < 0.05.

Abbreviations: CO, *Crataegus orientalis* ; CS, *Crataegus sinaica* Boiss.; CT, *Crataegus tanacetifolia* ; IC_50_, the half maximal inhibitory concentration.

A similar trend was observed for α‐glucosidase inhibition. Before digestion, CT again displayed the greatest inhibitory activity, followed by CS and CO. Although acarbose remained the most effective inhibitor, the inhibitory activity of CT approached that of the positive control. Simulated digestion significantly reduced α‐glucosidase inhibition in all samples; however, CS retained the greatest inhibitory activity after digestion, whereas CO exhibited the largest decline. These findings indicate that the compounds responsible for α‐glucosidase inhibition differ in their digestive stability among the investigated *Crataegus* species. Similarly, Huang et al. (2022) reported that flavonoid‐rich extracts from *Crataegus pinnatifida* exhibited inhibitory activity against both α‐amylase and α‐glucosidase, supporting the contribution of hawthorn phenolics to carbohydrate‐hydrolyzing enzyme inhibition. The reduction in enzyme inhibitory activity after digestion is consistent with the decreases observed in total phenolic content and individual phenolic compounds, suggesting that gastrointestinal conditions influence the stability of the phytochemicals involved in enzyme inhibition. Previous studies have shown that phenolic compounds may undergo hydrolysis, oxidation, or interactions with proteins, digestive enzymes, and bile salts during digestion, thereby reducing their inhibitory capacity (Cirkovic Velickovic and Stanic‐Vucinic 2018; Ketnawa et al. 2022). Differences among the three species are therefore likely associated with variations in phenolic composition rather than total phenolic concentration alone. Indeed, Liu et al. (2021) and de Paulo Farias et al. (2025) emphasized that the inhibitory potential of plant extracts depends largely on the type and structure of individual phenolic compounds. The positive association observed between rutin content and α‐amylase inhibitory activity in the present study agrees with previous reports describing rutin as an effective inhibitor of carbohydrate‐hydrolyzing enzymes through hydrogen bonding, hydrophobic interactions, and van der Waals forces with the enzyme active site (Martinez‐Gonzalez et al. 2019; de Paulo Farias et al. 2025). Likewise, Ahmed et al. (2024) highlighted that differences in flavonoid glycosides, phenolic acids, and procyanidins among *Crataegus* species contribute to variations in enzyme inhibitory activity. Previous studies have also reported positive relationships between phenolic content and the inhibition of α‐amylase and α‐glucosidase (Apostolidis and Lee 2010; Ramkumar et al. 2010). However, the present findings indicate that digestive stability, rather than initial phenolic concentration alone, is an important determinant of the inhibitory activity remaining after simulated gastrointestinal digestion. Although enzyme inhibition decreased following digestion, the persistence of measurable inhibitory activity, particularly in CT and CS, suggests that these species retain digestion‐resistant compounds capable of inhibiting carbohydrate‐hydrolyzing enzymes under the experimental conditions employed. Collectively, these findings indicate that simulated gastrointestinal digestion influences the stability of enzyme‐inhibitory constituents, underscoring the importance of assessing bioaccessibility in vitro as a first step toward understanding their potential physiological relevance, which should be confirmed through future in vivo studies.

### 3.7 Amino Acid Profile

The free amino acid profiles of the three *Crataegus* species before and after simulated gastrointestinal digestion are presented in Table 5. Because amino acid bioaccessibility is influenced by protein digestibility and gastrointestinal release, the amino acid composition of the IN fraction provides valuable information regarding the nutritional quality of plant proteins (Delgado‐Andrade et al. 2024).

**TABLE 5:** Amino acid composition results (mg/100 g) of hawthorn species in the undigested samples and IN phase.

Amino acid types (mg/100 g)	CT		CO		CS	
	Undigested sample	IN phase	Undigested sample	IN phase	Undigested sample	IN phase
l‐asparagine	98.27 ± 0.08^A,b^	68.41 ± 0.05^B,a^	158.76 ± 0.19^A,a^	55.70 ± 0.02^B,b^	136.38 ± 0.19^A,ab^	15.98 ± 0.24^B,c^
l‐threonine	8.49 ± 0.03^A,c^	2.86 ± 0.01^B,b^	10.47 ± 0.10^A,b^	3.56 ± 0.03^B,ab^	16.88 ± 0.04^A,a^	3.71 ± 0.02^B,a^
l‐serine	5.72 ± 0.01^A,a^	1.41 ± 0.03^B,a^	3.81 ± 0.02^A,b^	0.57 ± 0.02^B,b^	1.63 ± 0.01^c^	N.D.
l‐glutamic acid	189.79 ± 0.34^A,a^	112.61 ± 0.03^B,a^	167.79 ± 0.42^A,b^	38.97 ± 0.16^B,b^	155.56 ± 0.29^A,c^	20.67 ± 0.86^B,c^
l‐proline	1.37 ± 0.05^A,c^	0.21 ± 0.02^B,c^	4.70 ± 0.02^A,a^	0.45 ± 0.02^B,a^	2.61 ± 0.02^A,b^	0.30 ± 0.02^B,b^
l‐glutamine	18.41 ± 0.05^A,a^	4.19 ± 0.02^B,a^	6.84 ± 0.12^A,c^	0.50 ± 0.01^B,c^	11.47 ± 0.06^A,b^	2.64 ± 0.01^B,b^
l‐methionine	3.61 ± 0.03^A,b^	0.71 ± 0.02^B,a^	5.24 ± 0.02^A,a^	0.46 ± 0.01^B,b^	N.D.	N.D.
l‐leucine	8.70 ± 0.02^A,b^	0.96 ± 0.01^B,b^	2.55 ± 0.02^A,c^	0.32 ± 0.01^B,c^	11.65 ± 0.03^A,a^	1.44 ± 0.02^B,a^
l‐tyrosine	16.34 ± 0.04^A,b^	3.81 ± 0.03^B,b^	21.73 ± 0.01^A,a^	4.04 ± 0.09^B^	6.58 ± 0.01^A,c^	0.39 ± 0.04^B,c^
l‐phenylalanine	15.87 ± 0.03^A,b^	3.32 ± 0.03^B,ab^	9.30 ± 0.02^A,c^	2.52 ± 0.01^B,b^	22.72 ± 0.03^A,a^	3.62 ± 0.08^B,a^
l‐histidine	3.78 ± 0.03^A,b^	0.55 ± 0.02^B,b^	N.D.	N.D.	7.88 ± 0.04^A,a^	1.50 ± 0.08^B,a^
l‐lysine	17.44 ± 0.03^A,a^	5.21 ± 0.03^B,a^	8.60 ± 0.04^A,b^	1.81 ± 0.03^B,b^	N.D.	N.D.
l‐arginine	286.20 ± 0.44^A,a^	62.23 ± 0.66^B,a^	221.02 ± 0.83^A,b^	42.60 ± 0.07^B,b^	184.80 ± 0.42^A,c^	41.83 ± 0.27^B,b^
l‐valine	15.59 ± 0.05^A,b^	3.90 ± 0.02^B,b^	19.68 ± 0.05^A,a^	3.93 ± 0.04^B,b^	11.69 ± 0.05^A,c^	4.01 ± 0.03^B,a^
l‐alanine	8.32 ± 0.03^A,b^	1.49 ± 0.03^B,b^	4.51 ± 0.02^A,c^	0.45 ± 0.03^B,c^	18.59 ± 0.04^A,a^	3.10 ± 0.02^B,a^

*Note:* Different uppercase letters (superscript A, B) within the same line denote statistically significant differences among undigested sample and IN phase for each hawthorn sample (*p* < 0.05). Different lowercase letters within the same digestion phase (undigested or IN phase) indicate significant differences among *Crataegus* species (*p* < 0.05).

Abbreviation: N.D., not detected.

Fifteen free amino acids were analyzed, including essential and non‐essential amino acids. All amino acids were detected in CT, whereas l‐histidine was not detected in CO, and l‐methionine and l‐lysine were absent in CS in both digestion stages, while l‐serine was not detected in the IN fraction. Among the quantified amino acids, l‐arginine, l‐glutamic acid, and l‐asparagine were the predominant constituents in all three species, although their relative abundances differed significantly according to species and digestion stage (*p* < 0.05). Simulated gastrointestinal digestion significantly affected the amino acid profile of the hawthorn samples, with most amino acids showing lower concentrations in the IN fraction than in the undigested samples. The disappearance of certain amino acids after digestion, particularly l‐serine in CS, suggests that gastrointestinal processing influences the release and recovery of free amino acids. Despite these reductions, the predominance of arginine, glutamic acid, and asparagine was maintained after digestion, indicating that these amino acids were comparatively more stable during the simulated digestive process. The observed differences among the three *Crataegus* species demonstrate that amino acid composition is species dependent and is influenced by gastrointestinal digestion. Similar variability in amino acid composition has been reported for plant‐derived foods and has been attributed to genetic background, environmental conditions, and differences in protein composition (Delgado‐Andrade et al. 2024). The relatively higher abundance of l‐arginine, l‐glutamic acid, and l‐asparagine is noteworthy because these amino acids represent major nitrogen‐containing compounds in plant tissues. In particular, l‐asparagine plays a central role in nitrogen transport, storage, and remobilization during plant development, whereas l‐arginine and l‐glutamic acid are essential intermediates in nitrogen metabolism and amino acid biosynthesis (Gaufichon et al. 2010; Wang et al. 2025; Zhou et al. 2025). Changes in amino acid composition after digestion may also be associated with interactions between proteins and phenolic compounds. Previous studies have shown that flavonoid–protein interactions, through both covalent and non‐covalent mechanisms, can modify protein digestibility and influence the release of amino acids during gastrointestinal digestion (Kanakis et al. 2011; Larkin et al. 2007; Qie et al. 2021; Zhang et al. 2024). Such interactions may partly explain the reductions in several amino acids observed after digestion in the present study. Collectively, these results indicate that species‐dependent differences in amino acid composition persist after simulated gastrointestinal digestion, suggesting that the nutritional value of hawthorn proteins is influenced not only by their initial amino acid profile but also by the digestive stability of individual amino acids.

## 4 Conclusion

This study provides a comprehensive characterization of the nutritional composition, phenolic profile, antioxidant properties, enzyme inhibitory activity, amino acid composition, and mineral content of three *Crataegus* species, together with an evaluation of the effects of simulated gastrointestinal digestion on their phytochemical bioaccessibility and associated biological activities. The results demonstrated that gastrointestinal digestion markedly altered the phenolic composition and biological activities of hawthorn fruits, leading to significant reductions in total and individual phenolic compounds, antioxidant capacity, and α‐amylase/α‐glucosidase inhibitory activities. Despite the marked reductions observed during digestion, the persistence of measurable concentrations of several bioactive constituents in the bioaccessible fraction indicates that their digestive stability differs among the investigated *Crataegus* species. Distinct species‐dependent responses to simulated gastrointestinal digestion were observed among the investigated *Crataegus* species. CT displayed the highest bioaccessibility of total phenolics together with the greatest retention of reducing antioxidant capacity (FRAP and CUPRAC), despite showing the lowest ABTS and CUPRAC bioaccessibility. In addition, the three *Crataegus* species exhibited distinct free amino acid and mineral profiles, with l‐arginine, l‐glutamic acid, and l‐asparagine being the predominant amino acids across all species. Together with the observed differences in phenolic composition and bioaccessibility, these findings highlight the considerable nutritional and functional diversity among the investigated *Crataegus* species. Overall, the present study demonstrates that marked interspecific differences exist among the investigated *Crataegus* species with respect to their phytochemical composition, nutritional characteristics, and the bioaccessibility of their bioactive constituents following simulated gastrointestinal digestion. These findings not only enhance our understanding of the compositional and functional diversity of hawthorn fruits but also provide a scientific framework for identifying *Crataegus* genotypes with desirable characteristics for the development of functional food ingredients. While the present findings offer valuable insight into the fate of hawthorn phytochemicals under simulated gastrointestinal conditions, their physiological relevance should be confirmed through complementary cell‐based, bioavailability, and in vivo studies. Furthermore, the application of advanced LC–MS/MS‐based metabolomic approaches, together with in vivo investigations, will facilitate a more comprehensive understanding of the transformation, bioaccessibility, and biological significance of individual phytochemicals during digestion.

## Author Contributions

**Hatice Bekiroglu:** methodology. **Osman Sagdıc:** resources, supervision. **Gozde Kutlu:** investigation, writing – original draft, writing – review and editing, visualization, validation. **Kubra Feyza Erol:** conceptualization, investigation, funding acquisition, methodology, formal analysis, data curation, validation. **Fatih Tornuk:** supervision, resources.

## Funding

The authors have nothing to report.

## Conflicts of Interest

The authors declare no conflicts of interest.

## Data Availability

The datasets obtained during the current study are available from the corresponding author upon reasonable request.
